# Evaluating the Consistency Between Conceptual Frameworks and Factors Influencing the Safe Behavior of Iranian Workers in the Petrochemical Industry: Mixed Methods Study

**DOI:** 10.2196/22851

**Published:** 2021-05-27

**Authors:** Azita Zahiri Harsini, Philip Bohle, Lynda R Matthews, Fazlollah Ghofranipour, Hormoz Sanaeinasab, Farkhondeh Amin Shokravi, Krishan Prasad

**Affiliations:** 1 Department of Health Education, Faculty of Medical Sciences Tarbiat Modares University Tehran Iran; 2 Faculty of Medicine and Health The University of Sydney Sydney Australia; 3 Tasmanian School of Business and Economics University of Tasmania Private Bag 84, Hobart Tasmania Australia; 4 Work and Health Research Team, Faculty of Medicine and Health The University of Sydney Sydney Australia; 5 Health Research Center, Lifestyle Institute Baqiyatallah University of Medical Sciences Tehran Iran; 6 School of Business, School of Accounting Western Sydney University Sydney Australia

**Keywords:** safe behavior, petrochemical industry, conceptual frameworks, literature review, occupational health

## Abstract

**Background:**

Unsafe worker behavior is often identified as a major cause of dangerous incidents in the petrochemical industry. Behavioral safety models provide frameworks that may help to prevent such incidents by identifying factors promoting safe or unsafe behavior. We recently conducted a qualitative study to identify factors affecting workers' unsafe behaviors in an Iranian petrochemical company.

**Objective:**

The aims of this study were to (1) conduct a review of the relevant research literature between the years 2000 and 2019 to identify theoretical models proposed to explain and predict safe behavior in the workplace and (2) to select the model that best reflects our qualitative findings and other evidence about the factors influencing safe behaviors among petrochemical workers.

**Methods:**

This research used mixed methods. Initially, we conducted a qualitative study of factors that Iranian petrochemical workers believed affected their safety behavior. Four themes emerged from the semistructured interviews: (1) poor direct safety management and supervision; (2) unsafe workplace conditions; (3) workers’ perceptions, skills, and training; and (4) broader organizational factors. Electronic databases, including PubMed, Embase, Scopus, Google Scholar, EBSCOhost, and Science Direct, were then searched for eligible studies on models to explain and predict safe behaviors, which were published between the years 2000 and 2019. Medical subject headings were used as the primary analytical element. Medical subject headings and subheadings were then extracted from the literature. One researcher conducted the search and 3 researchers performed screening and data extraction. Then, constructs described in each study were assessed to determine which were the most consistent with themes derived from our qualitative analysis.

**Results:**

A total of 2032 publications were found using the search strategy. Of these, 142 studies were assessed and 28 studies met the inclusion criteria and were included in the review. The themes identified in the qualitative study most closely matched 3 scales included in Wu et al's model that measured safety behavior and performance, safety leadership, and safety climate in petrochemical industries. Poor direct safety management and supervision matched with safety leadership and its subscales; unsafe workplace conditions matched with safety climate and its subscales; workers' perceptions, skills, and training matched with safety performance and its subscales; and broader organizational factors matched with some subscales of the model.

**Conclusions:**

This is the first literature review to identify models intended to explain and predict safe behavior and select the model most consistent with themes elicited from a qualitative study. Our results showed that effective safety leadership and management and safety climate and culture systems are the most frequently identified factors affecting safe behaviors in the petrochemical industry. These results can further help safety researchers and professionals design effective behavior-based safety interventions, which can have a more sustainable and persistent impact on workers’ safety behaviors.

**Trial Registration:**

Iranian Registry of Clinical Trials IRCT20170515033981N2; https://www.irct.ir/trial/26107

**International Registered Report Identifier (IRRID):**

RR2-10.1186/s12889-019-7126-1

## Introduction

The International Labor Organization estimates that 1 worker in the world dies every 15 seconds because of occupational injuries and 160 workers develop work-related illnesses [1,2]. Workplace accidents not only cause occupational injuries and illness but also lead to financial losses for organizations [3]. Most behavior-based safety researchers consider that dangerous incidents are principally caused by workers acting unsafely or inappropriately and many studies have focused on worker behaviors that promote safety and prevent injuries [4]. Workplace safety is not solely explained by human error, and many other factors may contribute to this [5].

A substantial body of research indicates that organizational factors, including managers’ behavior and decisions, have a significant impact on safety-related attitudes and behaviors in industrial contexts [6]. Several studies, for example, indicate that safety performance is affected by leadership [6-8]. There is evidence that leadership and effective occupational health and safety management, particularly by direct managers and supervisors, is necessary to promote safe behavior [9,10]. Hald [11] noted that the role of leaders and managers typically involves various functions such as setting goals and monitoring and controlling workers’ performance. Other evidence indicates that broader organizational variables such as work intensification arising from increases in expected output or extended working hours are associated with poorer safety outcomes [12,13]. Another organizational factor is the contextual impact of safety climate [14,15]. Several studies have found a significant positive relationship between safety climate and safe behavior [16-18]. Safety climate is usually regarded as a subset of organizational climate that shapes workers’ behaviors through a coherent set of perceptions and expectations about an organization’s values and reward systems [19,20]. Various studies indicate that a poor safety climate leads to a reduction in compliance with safety procedures, which, in turn, causes an increase in the potential for workplace injuries and incidents [7,21-23].

Reason [24] describes 2 different ways to understand human errors at work: the individual (“person”) approach and the system approach. The first approach focusses on unsafe acts by workers, inappropriate ways of doing tasks that could result in a dangerous incident, for example, lack of or inappropriate use of personal protective equipment, operating equipment without qualification or authorization, or operating equipment at unsafe speeds [24]. The second approach focusses on unsafe working conditions or the state of the workplace system that could result in a workplace accident. Examples include defective tools, equipment or supplies, lack of emergency exits, and inadequate warning systems. Recent studies have placed importance on psychosocial conditions in policy and demonstrated the value of workers’ psychological well-being at work. Organizations that aim to concentrate on both physical and psychological factors together have safer working environments at lower risk of employee mental and physical health harm, and consequently, lead to positive workplace behaviors such as work engagement and safety incident reporting [25]. Many safe behavior studies have been based upon generic safety theories and models such as the Health Belief Model [26-29], the Theory of Planned Behavior [30-33], the Risk Perception Attitude Framework [34-36], and Social Cognitive Theory [37-39]. There is also a growing literature supporting the positive effects of safety behavior interventions on safety compliance and participation, injury rates, and near misses in various high-risk industries, including the oil, gas, and petrochemical industry [40-42].

A recent study by our research team [43] identified 4 sets of factors that workers believe discourage safe behaviors in an Iranian petrochemical company: (1) poor direct safety management and supervision, (2) unsafe workplace conditions, (3) workers' perceptions, skills, and training, and (4) broader organizational factors. The first aim of this study was to identify theoretical models proposed to explain and predict safe behavior in the workplace by reviewing relevant research studies published between the years 2000 and 2019. The second aim was to select the model that best reflects the results of our above-mentioned qualitative study [43] and other evidences on the factors influencing safe behaviors among petrochemical workers.

## Methods

### Study Design

The study protocol of this research has been published recently [44]. This study was a mixed methods research, which was carried out in 2 phases. In the first phase, semistructured interviews were conducted using a qualitative approach to gain detailed understanding of the factors associated with workers' unsafe behaviors in the petrochemical industry. In the second phase, models that have been applied to explain and predict safe behavior in the industrial settings were investigated. The findings of the first phase were matched with the constructs of the reviewed models to select a well-suited theoretical model.

### Qualitative Data Analysis

The interviews were conducted between May and July 2017 at a mutually convenient time and private areas at the participants’ workplaces. To obtain a broad cross-section of the worker opinions and experiences, multi-stage sampling was used. This approach involves a combination of 2 or more sampling techniques. By combining sampling methods at different stages of research, researchers can increase confidence that they are mitigating biases and engaging hard-to-reach, vulnerable participants [45]. In this study, purposive sampling was supplemented by snowball sampling to enhance recruitment. Purposive and snowball sampling approaches were selected because the research team considered the combination of the two was the most practical means to secure a representative sample of the company employees. The research team utilized purposive sampling, also known as judgmental sampling, to recruit particular interview subjects deliberately in order to provide important information and then snowball sampling to seek out further potential interviewees from the social network of the initial respondents [43]. Both techniques are used to achieve hard-to-reach participants in qualitative research studies [45].

Members of the company's Safety, Health, and Environment unit, who were not part of the research team, assisted with the sampling process. They invited workers, supervisors, and safety managers from various occupational groups working in the operations department and the maintenance and repair department who had experienced accidents and injuries or had witnessed colleagues’ accidents to participate in the study (purposive sampling). Workers were eligible to participate if they had worked in the petrochemical industry for at least 2 years. All workers in the petrochemical industry were males. During the interviews, respondents identified employees who had information about workplace accidents in the company and were key informants (snowball sampling). These employees were also invited to participate in the study. Before the start of each interview, a member of the safety staff introduced the participant to the first author, who provided clear verbal information about the study [43].

Data saturation is a criterion that is used to justify adequate sample sizes in qualitative studies. Data saturation is reached when the final interviews do not reveal any new themes or introduce new elements of an existing theme. When saturation is achieved, additional interviews only generate redundant data rather than novel findings. A total of 20 interviews were conducted before saturation was reached. The 20 participants consisted of workers, supervisors, and safety staff members. For the analysis of the responses from Iranian petrochemical workers [43], conventional content analysis, described by Graneheim and Lundman [46], was used to interpret the content of the interview transcripts through a systematic classification process involving coding and identifying themes [47]. A team of 6 coders (4 in Iran and 2 in Australia) reviewed the transcripts and conducted analysis in both languages. Open coding was carried out to allow codes to emerge from the qualitative data and avoid codes based on preconceptions of the authors. Codes were repeatedly discussed and revised by the authors to achieve consensus and memos written to explain the analysis [48]. To increase interrater coding reliability, only the codes and themes that were validated by at least 2 of the 3 coders (the first author, an Iranian and 2 Australian authors) were included in the results. Immersion in the data was an important first stage in the analysis process during which transcripts were read and reread many times to ensure familiarity with the data. Repeated reading and rereading of transcripts without coding helped identify emergent themes from the data without losing the connections between key concepts and their context.

Content analysis was performed using MAXQDA (version 2018) software (VERBI Software GmbH) to facilitate and document the coding process and retrieve codes afterwards. While software can assist researchers in organizing qualitative data, computer software for qualitative analysis do not analyze data and the researcher makes decisions about the coding participants’ responses and the relationships between codes, coding categories, and broader themes. MAXQDA allows the researcher to upload raw data such as transcribed interviews that can be then coded and cross-referenced in ways that facilitate organizing the data for easy retrieval.

### Literature Search Sources and Strategy

A literature search of publications in academic journals and conference papers covering the period 2000-2019 was conducted using the following web-based databases: PubMed, Embase, Scopus, Google Scholar, EBSCOhost, and Science Direct. A review of the literature revealed a lack of consensus among research studies regarding factors that discourage safe work behaviors and the risk of incidents occurring in industrial settings. The gap in the literature was identified in the 2000s. These eligibility dates were chosen to provide a sample of studies, including the constructs to explain and predict safe behavior using models. The reference lists of the included studies were also searched to identify additional relevant studies. We applied a predefined search strategy by using free terms and medical subject heading terms. Terms referring to safety were combined with OR, terms referring to safe behavior were combined with OR, and terms referring to both were combined with AND. The following free terms were used in all electronic databases: safety, behavior, worker, and workplace. The following medical subject heading terms were employed: safety, safety behavior, safe work behavior, behavior-based safety, workers' behavior, safety models, and workplace safety. The references provided in the publications identified were also examined. When full-text publications were not available directly from electronic databases, the authors of the studies were contacted and copies of their articles were requested. The search results were updated using Google alerts.

The publications were filtered using a set of inclusion and exclusion criteria. Inclusion criteria were that the publication described the (1) development of a theoretical model as a tool to assess safe work behavior, (2) application of a theoretical approach and method that had been used to assess workplace safety, or (3) definitions used to describe and evaluate safe work behaviors. Publications that did not describe the development or application of a safe work behavior model were excluded. Non-English papers, conference abstracts, literature reviews, editorials, commentaries, letters to the editor, theses, and full texts that were not accessible were also excluded.

### Investigation Models

Publications were reviewed to identify theoretical models that have been used to explain and predict safe behavior in the petrochemical industry or other industrial settings. The key constructs in the models were then evaluated for consistency with the themes identified in our qualitative study of workers in the Iranian petrochemical industry [43]: *poor direct safety management and supervision*; *unsafe workplace conditions*; *workers' perceptions, skills, and training*; and *broader organizational factors*. The model including constructs that were the most consistent with the qualitative findings was then identified.

## Results

### Study Selection

**Figure 1:**
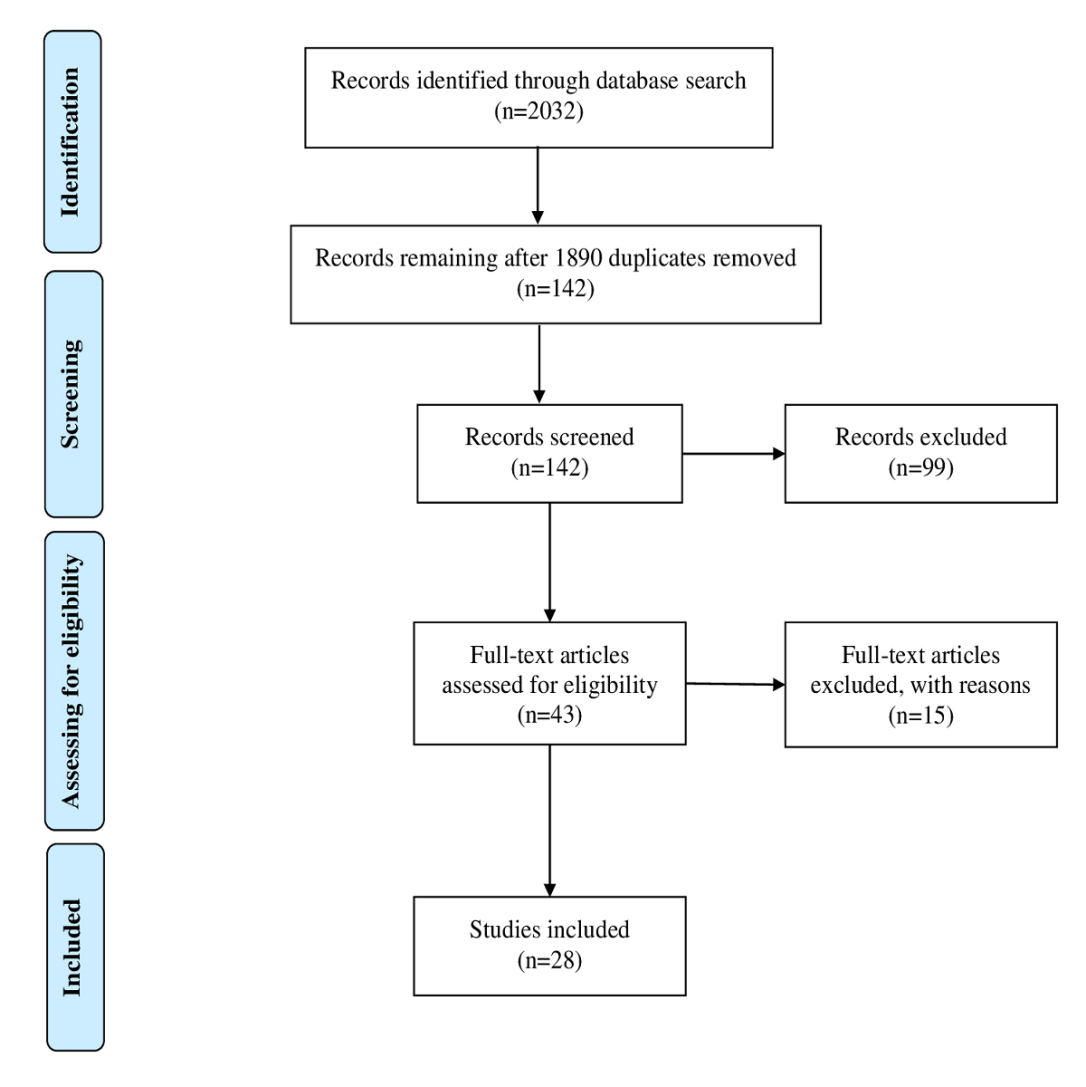
Flow diagram of the search results and the study selection process using the PRISMA template.

This review was conducted in accordance with the PRISMA (Preferred Reporting Items for Systematic Reviews and Meta-Analyses) statement [49]. A flow diagram describing the process for reviewing the studies is provided in Figure 1. In total, 2032 publications were retrieved from the databases listed in the Methods section. Duplicate publications were removed, and 142 (84 academic journal articles, 55 reports and other publications, and 3 PhD theses) were screened by reading the title, abstract, and key words. By using the inclusion and exclusion criteria, 99 studies were excluded from the review, leaving 43 studies eligible for full-text review. During this review, 15 publications were excluded, because they did not meet the inclusion criteria. Ultimately, 28 studies were included in this review. The themes, categories, and codes that emerged from the content analysis of the semistructured interviews are listed in Table S1 of Multimedia Appendix 1. An overview of the final chosen set of publications eligible for review and the constructs used in each of them is provided in Table 1. All study selection processes were performed using EndNote X8.1 (Clarivate Analytics).

**Table 1:** Description of the included studies (listed by the year of publication) and the constructs used in each of them.

Study	Country of origin	Industry context	Constructs included in the model
Griffin and Neal (2000) [50]	Australia	A range of manufacturing and mining organizations	Manager values, safety inspections^a^, personal training^a^, safety communication^b^, safety knowledge^a^, safety compliance^c^, safety participation^c^
Brown et al (2000) [51]	United States of America	Steel industry	Safety hazards, safety climate^b^, pressure^b^, cavalier attitudes^d^, safety-efficacy^c^, safe behavior^c^
Hong et al (2004) [52]	Taiwan	Petrochemical industry	Training courses^a^, workers’ cognition and attitude^d^, behavior and normative belief, behavior attitude^d^, subjective norm, behavior^c^
Seo (2005) [53]	United States of America	Grain industry	Perceived safety climate^b^, perceived hazard level, perceived work pressure^b^, perceived risk, perceived barriers, unsafe work behavior^c^
Pousette et al (2008) [54]	Sweden	Construction	Safety climate^b^, safety motivation^b^, safety knowledge^a^, self-rated safety behavior^c^
Larsson et al (2008) [55]	Sweden	Construction	Psychological climate^b^, job situation, workplace commitment^b^, safety motivation^b^, safety knowledge^a^, safety behavior^c^
Zhou et al (2008) [56]	China	Construction	Safety climate^b^, safety management^a^, management commitments^a^, safety attitudes^c^, workmate’s influence, employee’s involvement^b^, personal experience, safety knowledge^a^, education experience^a^, work experience^a^, drinking habits, safety behavior ^c^
Lu and Yang (2010) [57]	Taiwan	Container terminal companies	Safety motivation^b^, safety policy, safety concern, safety compliance^c^, safety participation^c^
Martínez-Córcoles et al (2011) [6]	Spain	Nuclear power plant	Empowerment leadership^a^, safety culture^d^, safety climate^d^, safety behaviors^c^
Wu et al (2011) [42]^e^	Taiwan	Petrochemical company	*Safety leadership*^a^: safety coaching^a^, safety caring^d^, safety controlling^a^ *Safety climate*^b^: workers’ commitment to safety^b^, perceived risk^b^, emergency response^b^ *Safety performance*^c^: safety inspection^a^, accident investigation^c^, safety training^d^, safety motivation^b^
Li et al (2013) [58]	China	Oil company	Job demands^b^, job resources, emotional exhaustion, safety compliance^c^, safety outcomes
Qinqin et al (2014) [59]	China	Petrochemical industry	Hazardous materials, production process, equipment condition^a^, environmental safety and health^b^, vulnerability of receptor^c^
Shin et al (2015) [60]	South Korea	Construction	Management values, safety climate^b^, stress response^b^, safety motivation^b^, safety knowledge^a^, safety behavior^c^
Wu et al (2015) [61]	China	Railway construction	Safety leadership^a^, design and planning for safety, preconstruction hazard inspection^a^, construction process safety, emergency preparedness^b^, management auditing and organizational learning, safety performance^c^
Azadeh et al (2015) [62]	Iran	Petrochemical plant	Physical factors of workplace^b^, environmental features and issues^b^, management systems and control^a^, individual protection tools^a^, workplace safety actions, on-the-job training^a^, passing ways, monitors and displays^a^, muscular and skeletal disorders^c^, anthropometric features and issues, job characteristics, layout feature and issues, job and environmental satisfactions, overall health, safety, and environment management and performance^a^, mental workload and stress^b^
Alshahrani et al (2015) [63]	Saudi Arabia	Petrochemical industry	Safety culture^d^, safety attitudes^b^, safety and health requirements to circumvent accidents at workplace, safety behavior^c^, safety performance^c^
Wang et al (2016) [64]	China	Construction	Personal subjective perception^b^, work knowledge and experiences^c^, work characteristics, safety management^a^, workers’ safety risk tolerance
Zhang et al (2016) [65]	China	Coal mining	Safety management agency^a^, rules and regulations of safety production^a^, defect of technology and design^b^, lack of safety education and training^a^, incomplete or poor execution of rules and regulations, rules and regulations and inspection^a^, safety culture^d^, operator error, venturing into dangerous places, protections, device signal deficiencies^a^, equipment, facilities and tools^a^, poor workplace environment^b^
Petitta et al (2017) [14]	Italy	Manufacturing, construction, transportation, military, energy, health care, and distribution/service	Safety compliance^c^, supervisor enforcement^d^, organizational safety climate^b^, organizational safety culture^d^
Zaira and Hadikusumo (2017) [66]	Malaysia	Construction	Management safety intervention^a^, human safety intervention, technical safety intervention, safety behavior^c^
Jafari et al (2017) [67]	Iran	Petrochemical company	Management commitment^a^, workers’ empowerment, communication^b^, blame culture, safety training^a^, safety supervision^a^, interpersonal relationship^b^, continuous improvement, reward system^b^, job satisfaction
Razmara et al (2018) [27]	Iran	Taxi stations	Perceived susceptibility, perceived severity, perceived benefits^c^, perceived barriers^c^, self-efficacy^c^, cues to action, safe driving behavior^c^
Nioi et al (2018) [30]	United Kingdom	Construction	Behavioral beliefs^c^, normative beliefs, control beliefs, attitudes toward the behavior^d^, subjective norms, perceived control^c^, behavioral intention^c^, behavior^c^
Hald (2018) [11]	China	Electronics industry	Safety climate^b^, safety hazards, experience with safety and health problems^c^, pressure^b^, employees’ knowledge of the factory^a^, cavalier attitudes toward safety^b^, safety efficacy^c^, safe workplace behavior^c^
Zhang et al (2018) [68]	China	Petrochemical enterprise	Personnel training^a^, fire facilities, fire management, technical level
Newaz et al (2019) [19]	Australia	Construction	Management safety commitment^a^, supervisor safety behavior^a^, coworker safety behavior ^b^, psychological contract of safety^b^, worker safety behavior^c^
Gao et al (2019) [41]	China	Oil industry	Leadership/management commitment^a^, organizing responsibilities/procedures, communication and coordination^b^, safety training^a^, inspection and monitoring^a^, employee involvement^b^
Wang et al (2019) [69]	China	Coal mining	Workers’ characteristics, workers’ perception of safety, working pressure^b^, leader’s attitude in meeting, inspectors’ quality^a^, management system’s integrity^a^, management system’s stringency^a^

^a^Matches with the theme of poor direct safety management and supervision in our qualitative study.

^b^Matches with the theme of unsafe workplace conditions in our qualitative study.

^c^Matches with the theme of workers’ perceptions, skills, and training in our qualitative study.

^d^Matches with the theme of broader organizational factors in our qualitative study.

^e^The italicized constructs identified in the model most closely matched those identified in our qualitative study.

### Study Characteristics

Eleven (39%) out of 28 studies were conducted in a single industry (steel [51], grain [53], container terminal companies [22], nuclear power [6], oil [41,58], railway construction [61], coal mining [65,69], taxi stations [27], electronics [11]), and 2 studies (7%) included multiple industries [50,57]. Industries attracting the most studies were the construction (8/28, 29%) and petrochemical (7/28, 25%) industries. Ten studies were conducted in China (36%). Many regulations, occupational health and safety laws, and documents have been issued to help industries establish a positive safety culture in China. According to the Unemployment by Country 2021 [70], 23 out of 28 studies (82%) were conducted in countries with low unemployment (Malaysia, South Korea, Taiwan, China, United States, the United Kingdom, Australia, Saudi Arabia, and Sweden) and 5 studies (18%) were conducted in countries with high unemployment (Italy, Iran, and Spain).

### Contributing Factors

Elements of the models presented in the 28 selected studies were evaluated for consistency with the 4 factors identified in our previous study: *poor direct safety management and supervision*; *unsafe workplace conditions*; *workers' perceptions, skills, and training*; and *broader organizational factors*. All the emergent themes, categories, and codes matched up directly with each of the constructs included in the models in the general industrial settings and petrochemical industry. Concept matches in each of the studies are highlighted in Table 1 by labelling each match as “a,” “b,” “c,” or “d” to indicate which of our 4 contributing factors it corresponds with. Based on the number of these matches, the model most consistent with the 4 contributing factors was identified.

The *poor direct safety management and supervision* theme combines 2 categories, namely, ineffective safety system and poor safety monitoring. Concepts in the reviewed models that correspond with these categories have been labelled as “a” (Table 1). Of the 28 studies evaluated, the model constructs of 20 (71%) studies were matched with categories and codes of theme “a” [6,11,19,41,42,50,52,54-56,59-62,64-69].

The *unsafe* *workplace conditions* theme consists of 2 categories: unsafe physical environment and unsafe psychological environment. The codes of these categories have been matched with concepts in the reviewed models by using the character “b” in Table 1. Of the 28 assessed studies, constructs included in the models of 22 (78%) studies were matched with categories and codes of theme “b” [11,14,19,41,42,50,51,53-65,67,69,71].

The *workers' perceptions, skills, and training* theme consists of 2 categories: workers not skilled enough to deal with safety issues and active errors. The codes of these categories have been matched with concepts in the reviewed models by using the character “c” in Table 1. Of the 28 assessed studies, 23 (82%) found constructs included in the model and matches with categories and codes of theme “c” [6,11,14,19,27,30,42,50- 64,66].

The *broader organizational factors* theme includes unsafe management culture and organizational impact on workers’ safety categories. The codes of these categories have been matched with concepts in the reviewed models by using the character “d” in Table 1. Constructs applied in 8 (28%) of the 28 studies included in the literature review were matched with categories and codes of theme “d” [6,14,30,42,51,52,63,65].

### Selection of the Theoretical Model

The purpose of reviewing the models of safe work behaviors was to (1) identify constructs included in the selected models and (2) identify the model that included constructs most consistent with the findings of the preceding qualitative study of Iranian petrochemical workers’ perceptions of factors affecting safe work behaviors [43]. The constructs identified in the model described by Wu et al (see Table 1 [42]) most closely matched those identified in our qualitative study. Wu et al [42] proposed a theoretical model relating to safety behaviors in a petrochemical company and explored 3 major factors, namely, *safety leadership, safety climate*, and *safety performance*. Safety leadership consists of 3 subscales: *safety coaching, safety caring, and safety controlling*. Safety climate also consists of 3 subscales: *workers’ commitment to safety, perceived risk, and emergency response*. Safety performance consists of 4 subscales: *safety inspection, accident investigation, safety training, and safety motivation*. The constructs described by Wu et al [42] were well matched to the contributing factors identified in our qualitative study: safety leadership and its subscales matched with poor direct safety management and supervision; safety climate and its subscales matched with unsafe workplace conditions; safety performance and its subscales matched with workers' perceptions, skills, and training; and codes from several subscales matched with broader organizational factors.

## Discussion

### Principal Findings

This study evaluated the consistency between 28 theoretical models proposed to explain and predict safe behaviors in industrial settings and qualitative findings of our previous study examining the factors that petrochemical workers perceived to affect safe behaviors. The first aim of this study was to identify the theoretical models that were developed to explain and predict safe behavior in both the petrochemical industry and general industrial settings. The second aim was to select the model that corresponds most closely with our qualitative findings. The majority of the included studies were found to focus on some aspects of our qualitative data. Most of the studies were conducted in various industrial domains. Our findings indicate that the key elements of the model described by Wu et al [42] corresponded most strongly with the themes derived from our qualitative interview study. Several of the other models identified in the review also included elements that corresponded closely with the themes identified in our interview study.

### Comparison With Previous Studies

Based on the findings from our review, the safety concern of managers and supervisors was identified as the most key factor affecting the workers’ risk perception and their understanding of safety issues [19,42,56,62,72]. In addition, supervisors’ safe behaviors such as regular safety inspection, motivating and supporting the subordinates, and providing resources for appropriate training of the workforce can motivate safety performance, encourage workers’ participation as well as reporting potential incidents and unsafe behaviors [41,50,61,64,69,71]. Managers have a crucial role in the success of workplace health promotion activities and changing employee health behavior. Managers and supervisors are able to create a safe organizational climate and positively influence employees’ healthy and safe work behavior by providing necessary resources for planning, implementing, and evaluating workplace health promotion interventions. Supporting workplace health promotion programs can enhance the engagement of the employees and benefit both organizations and employees in the long run [73]. These findings are consistent with the *poor direct safety management and supervision* theme of our qualitative study. The included studies assessed the relationship between safety climate and workers’ perceptions of safety issues and various aspects of safety-related behavior. These studies examined work safety climate and aspects of working conditions and their associations with occupational safety and work-related injuries among various workplace settings [11,42,51,53,69]. They focus mainly on improving working conditions and its organizational and psychological aspects such as perceived work pressure, emergency response, physical and psychosocial hazards at work, job demands, physical factors of workplace, mental workload and stress, and defect of technology and design [42,53,58,62,65,72]. A Korean study reported that working conditions are important key factors that could influence workers’ behavior at the workplace. The employment status of workers impacts the organizational commitment and safety performance. Even within the same organization, workers in different employment statuses are treated differently. Because of the health inequalities of temporary employment such as workers’ compensation and welfare programs, employment status affects workers’ health and causes disparities in safety, which is compounded by unsafe workplace settings. In fact, occupational injury rates for part-time and temporary contract workers are significantly higher than those for regular and permanent workers in the same occupation [74]. These results support our qualitative findings related to *the unsafe workplace conditions* theme. According to the review of 28 studies, adequate and appropriate job training, workers’ perception of risk, and their knowledge of health and safety issues were negatively correlated with occupational accident rates [50,52,71]. Workers' skills and perceptions of their own behavior plays a significant role in producing better safety outcomes [27,30,42,64,75]. These findings are also consistent with the *workers' perceptions, skills, and training* theme of the qualitative study. The findings of the included studies also focused on the importance of management culture and organizational impact on workers' safety. These findings highlight that workers’ cognition and attitude, safety culture, and prioritizing safety can influence workers to adopt positive behavioral intentions toward safety at workplace [6,14,42,52,56,63,72,76]. These findings also support the fourth theme of our qualitative findings: *broader organizational factors*.

Nixon and Braithwaite [77] in their detailed qualitative investigation suggest that a well-developed conceptual model can be employed to train employees, manage their progress, and develop high work performance culture. Wu et al’s [42] model suggests that 2 important prior causes greatly affect safe behaviors and performance: safety leadership and safety climate. In this context, the role of managers and supervisors in shaping subordinates’ safe behaviors is likely to be considerably greater than that of managers and supervisors in work settings with routine production processes [78]. Consistent with our qualitative findings, the results of a sample of 103 industrial organizations located in Spain indicated that supervisor enforcement and managers' commitment to safety is significantly related to workers’ safety compliance [79]. Supervisors have the most frequent contact with employees and workers among the hierarchical levels of an organization and are directly responsible for guaranteeing safety performance at the workplace. Managers’ responses to safety are a key determinant in the creation of subordinates’ beliefs about the importance of safety to the work settings [80,81]. As expected, a positive safety culture will be developed when managers commit to the priority of safety [41]. In addition, workers perceive that the role of both the managers and supervisors in combination with their safety commitments enables workers to develop a mutual obligation with them and these obligations will lead to safer behavior of workers [82].

The findings of our qualitative study indicated that unsafe workplace conditions may be a particularly strong influence on whether work is done safely or not. Wu et al [42] defined safety climate as “employees' perception, attitudes, beliefs, and values of safety of an environment or organization, which is affected by personal and organizational factors, and affects employees’ safety performance.” The relationship between safety climate and safe work behavior has been well established in safety research, and safety climate has been identified as a critical indicator for enhanced safety, which has been linked to increased safe behaviors and decreased injury severity in industrial settings [83-85]. Safety climate is therefore related to how workers perceive organizational priorities in their workplace and has a major role in motivating workers to work safely [86]. Safety climate is indicated by the perceptions of norms and actions that help to prevent unsafe acts [20]. Furthermore, Beus et al [87] reported that a supportive safety climate is associated with higher rule compliance and fewer work-related injuries. A positive organization’s safety climate provides workers with cues and vital information regarding the extent to which safe behaviors are valued, supported, and rewarded in the workplace [88]. Studies have shown that safety climate scores are significantly predictive of worker safety attitudes, safety compliance and performance, workplace accidents, injuries, near misses, safety knowledge, and safety motivation [89-91].

Another factor identified in our qualitative study was workers’ perceptions, skills, and training. Occupational hazards and safety performances are affected by factors, including workers’ safety attitude and knowledge [42]. Findings indicate that workers with more knowledge of the products, work environment, and objectives of the organization demonstrated a higher level of safe behaviors in their contexts as compared to their ignorant colleagues [92]. Workers’ knowledge, skills, and competence with regard to safety are the required content of safety training [93,94]. Workers who do not fully understand the safety and health instructions that are related to their jobs tend to experience higher accident rates. In addition, owing to differences in the education level, safety training should be provided separately according to workers’ education levels and ages. Therefore, safety training should be designed in accordance with the requirements for workers to be aware of safety at work [75,95]. Korkmaz and Park [75] also agreed that workers who are familiar with their job tasks could help by being involved in the risk assessment in the workplace. Researchers [96,97] found that organizations can have low injury and accident rates when they predict and implement practical safety training regularly.

In Wu et al’s model [42], safety performance reflects the workers’ perceptions, skills, and training. Safety leadership has been associated with safety management and supervision, in general. Further, the dimensions of safety climate (workers’ commitment to safety, perceived risk, and emergency response) are consistent with categories and codes of the unsafe workplace conditions theme. Since our qualitative findings align with the dimensions of the established model by Wu et al [42], we evaluate this model as applicable in order to design educational interventions for petrochemical workers. Technical intervention safety practices have a positive effect on safe work behaviors. In addition, the management safety intervention plays a significant role in the implementation of safety practices. Therefore, this model provides some guidance to industrial companies to better focus on specific safety intervention practices that improve workers’ safe behaviors and their safety awareness to work safely.

### Implications for Research and Practice

The current literature search identified 28 studies that served as examples for the translation of a safety model into intervention efforts, which can guide workplaces to improve their safety conditions and reduce accident rates. When reviewing the models in the 28 selected studies, the main feature of the model was assumed from the assessment of general levels of safety and major components of conceptualizing safety (eg, safety management, safety climate) to special and detailed latent hazard conditions such as levels of organizational support, and risk perceptions might be seen to imply that safety models are seen as ways to assess the wider and bigger picture of how safety promotion might work in industrial contexts.

### Limitations of This Study

This study enhances understanding of the factors affecting safe work behavior and highlights directions for further research. However, some important limitations should be recognized. A key limitation, which was difficult to avoid, is the exclusive focus on published research. This review included studies published in peer-reviewed journals. Although this was done to provide a high quality of evidence and findings, the criteria excluded a number of potentially valuable research and industry reports or unpublished studies. Evidence suggests that use of workplace safety models may be underreported. The studies identified, which were drawn from a variety of settings (eg, petrochemical, construction, oil and gas), indicated that safety models are widely used by organizations that are eager to develop better understanding of safety risks in their workplaces. A key weakness of the safety model approach may be that results obtained at one point in time may not prove to be repeatable at another. The studies reviewed in this paper do not allow firm conclusions to be drawn about the reliability, validity, and overall robustness of using safety models in practice. Deeper investigation into these issues would be a valuable focus for future research. The aim of the improvement plan is to have a better safety status by making suggestions for the Iranian petrochemical industry for workers. However, this may be applied in other countries. Nevertheless, this subject should be studied more for other industrial settings and countries in order to reach a more generalized result.

### Conclusions

This study is the first, to the best of our knowledge, to examine the key variables in theoretical frameworks designed to explain safety behaviors in industrial settings, identify potentially relevant theoretical models, and evaluate the suitability and applicability of the models identified to explaining the safety of petrochemical workers based on independent qualitative findings about the factors that discourage safe work behaviors. The findings indicate growth in terms of the use of safety models to assess workers’ safe behaviors and significant variation in the ways in which they are used and reported in the safety literature. For safety researchers and practitioners, the results are important because the models provide guidance on how workers may be influenced to work more safely. By identifying the conditions in which workers can be encouraged to change unsafe behaviors to safe ones, integrated safety intervention models can provide a valuable tool for enhancing safety performance. Lastly, this study has implications for leadership at both the supervisory and management levels by identifying the effects of supervisor' behaviors and safety climate as determinants of safety performance. Taken as a whole, our findings encourage a holistic approach that takes into account both safety management and climate to comprehensively understand the individual and contextual factors that shape safe work behaviors in the petrochemical industry. It is important that future theoretical and conceptual frameworks address the inconsistencies identified in this study to enable the adoption and replication of safe behavior interventions in industries, thereby preventing workplace injuries and fatalities and making workplaces healthier and safer.

## Appendix

Multimedia Appendix 1 Themes, categories, and codes from the qualitative analysis.
