# Supplementary material for: Evaluating the Consistency Between Conceptual Frameworks and Factors Influencing the Safe Behavior of Iranian Workers in the Petrochemical Industry: Mixed Methods Study
Source: JMIR Public Health Surveill. 2021 May 27;7(5):e22851. doi: 10.2196/22851 (PMC8193472; doi:10.2196/22851)
Supplement: Multimedia Appendix 1 [file publichealth_v7i5e22851_app1.doc]

**Table S1.** Classiﬁcation of themes, categories and codes derived from the content analysis of interview responses from Iranian petrochemical workers.

| Theme | Category | Code |
| --- | --- | --- |
| Poor direct safety management and supervision | Ineffective safety system | - Inadequate safety training for workers and safety staff - Inappropriate quality and design of personal protective equipment - Managers not carrying their safety management role effectively - Sub-standard or inappropriate safety equipment promotes accidents - Supervisors not emphasizing and prioritizing safety - No separate allocation of funds to improve safety |
|  | Poor safety monitoring | - Managers' lack confidence to deal with safety hazards or issues - Safety officers not enforcing safety practices and lacking experience and authority - Inadequate number of safety officers on site - Irregular safety inspections - Contractors not prioritizing safety equipment and training |
| Unsafe workplace conditions | Unsafe physical environment | - Excessive noise impairing concentration - Use of worn-out and defective equipment - Working in high temperatures |
|  | Unsafe psychological environment | - Work-related fatigue - Excessive workloads - Delayed salary and wage payments reducing safety incentives - Poor social working environment - Inadequate pay and financial detract from focus on safe behaviour - Low safety motivation - Little encouragement for workers to contribute to safety - Work-related stress - Separation from family - Low level of organizational commitment |
| Workers' perceptions, skills and training | Workers not skilled enough to deal with safety issues | - Lack of experience and skills in dealing with hazards. - Taking greater risks when doing common tasks - Need for more sharing of previous experiences with hazards - Hazards becoming ‘normalized’ over time - Inadequate safety orientation for new workers - Use of untested work practices |
|  | Active errors | - Workers distracted by making errors - Not seeking help when minor incidents occur - Workers ignoring safety instructions for machinery - Low level of safety efficacy - Unrecognised health conditions contributing to errors |
| Broader organisational factors | Unsafe management culture | - Prioritizing work outcomes over safety - Management purchases low-quality safety products and equipment - Condescending safety supervision and bullying |
|  | Organisational impact on workers' safety | - Lack of attention to workers' emotional and mental needs - Lack of organizational safety training at appropriate levels - Workers underestimating routine hazards - Poor organisational safety culture influencing workers’ behaviour - Inadequate staffing - Incidents may occur even when workers behave safely |

NOTE. Every category is described using codes extracted from the interviews.
